# Research Progress on the Application of Mass Spectrometry Imaging Technology in Cerebral Disease

**DOI:** 10.3390/life16010168

**Published:** 2026-01-20

**Authors:** Yao Qiao, Jie Yin, Shuyu Lu, Lihui Yin

**Affiliations:** 1 National Institutes for Food and Drug Control, Beijing 102629, China; qiaoyao@nifdc.org.cn (Y.Q.); yinjie@nifdc.org.cn (J.Y.); 2 State Key Laboratory of Drug Regulatory Science, Beijing 102629, China; 3 College of Pharmacy, China Pharmaceutical University, Nanjing 210009, China; lushuyu0803@163.com

**Keywords:** mass spectrometry imaging, brain diseases, lipid metabolism, spatial omics, neurodegenerative disease, psychiatric disorder, cerebrovascular event

## Abstract

Mass spectrometry imaging (MSI) is an innovative analytical technique that integrates chemical analysis with spatial localization, enabling label-free, in situ detection and visualization of diverse biomolecules within tissue sections. This review summarizes the recent advances in the application of MSI to neurological disorders, with a focus on Parkinson’s disease, Alzheimer’s disease, schizophrenia, and traumatic brain injury. Studies have demonstrated that MSI can delineate the spatial heterogeneity of disease-related molecules—such as neurotransmitters, lipids, and metabolites—thereby providing new perspectives for understanding the pathological mechanisms of neurodegenerative and psychiatric diseases. Platforms including MALDI-MSI and DESI-MSI have been effectively employed for visualizing drug distribution, characterizing lipid metabolic pathways, and identifying spatial biomarkers. Although challenges remain in quantitative accuracy, spatial resolution, and the detection of low-abundance molecules, advances in high-resolution mass spectrometry, single-cell-level imaging, and multi-omics integration are expected to further enhance the utility of MSI in the investigation of brain diseases.

## 1. Introduction

**Figure 1:**
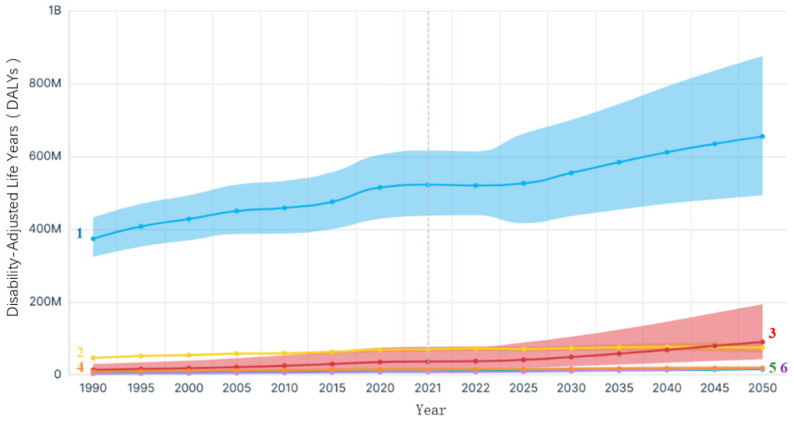
Predicted Trends in the Global Burden of Brain Disorders, all genders, global (1990–2050). 1 All brain disorders; 2 Ischemic stroke; 3 Alzheimer’s disease and other dementias; 4 Schizophrenia; 5 Brain and nervous system tumors; 6 Parkinson’s disease.

Cerebral diseases—including neurodegenerative disorders, psychiatric conditions, cerebrovascular events, traumatic brain injury, and intracranial tumors—represent a major global health challenge, contributing substantially to long-term disability, mortality, and socioeconomic burden [1]. According to data from the Brain Health Atlas, disability-adjusted years attributable to brain disorders have steadily increased since 1990, and projections indicate that the associated disability-adjusted life years (DALYs) may exceed 800 million by 2050. In 2021, approximately 520 million individuals worldwide were affected by various brain disorders, with the resulting DALYs and economic losses far surpassing those of many other chronic diseases (Figure 1).

The brain, as a highly complex and heterogeneous organ, maintains its higher-order functions through the precise regulation of molecular networks—including proteins, lipids, metabolites, and neurotransmitters—that act synergistically across distinct brain regions, neural circuits, and even at the single-cell level. However, the onset and progression of various brain disorders are consistently accompanied by marked alterations or abnormal remodeling in the spatial distribution and abundance of these essential biomolecules [2,3]. Traditional research approaches face inherent limitations in capturing molecular spatial information. Macro-scale omics analyses based on tissue homogenates, for example, provide global molecular profiles but completely eliminate critical spatial context within the tissue microenvironment [4,5]. Conversely, antibody-mediated imaging techniques such as immunohistochemistry offer excellent spatial resolution yet are restricted to predefined targets, limiting their capacity for unbiased exploration of unknown biomarkers or complex molecular networks [2,4]. This methodological bottleneck in obtaining molecular spatial heterogeneity has substantially constrained our ability to elucidate the underlying pathophysiological mechanisms of brain disorders and their microenvironmental interactions.

The emergence of mass spectrometry imaging (MSI) has provided a revolutionary solution to this challenge. As a label-free, high-throughput [6] analytical strategy, MSI enables direct in situ mass spectrometry scanning on thin tissue sections, simultaneously acquiring mass spectral signals and two-dimensional (or even three-dimensional) spatial distribution information for hundreds to thousands of endogenous or exogenous molecules within the tissue. Its core advantage lies in revealing unique expression patterns of multiple biomolecules within the tissue’s native environment in a non-targeted manner, while preserving the integrity of the tissue’s microscopic architecture. These capabilities make MSI a powerful tool for elucidating molecular spatial heterogeneity in brain tissue and for tracking key molecular events during disease progression—such as abnormal protein deposition [7], lipid spatial distribution [8,9] metabolite pathway alterations [10], and identifying spatially resolved biomarkers [7].

In recent years, driven by continuous innovations in multiple technical platforms—including Matrix-Assisted Laser Desorption/Ionization Mass Spectrometry Imaging (MALDI-MSI), Desorption/Ionization Electrospray Mass Spectrometry Imaging (DESI-MSI), and Secondary Ion Mass Spectrometry Imaging (SIMS-MSI)—MSI has achieved significant breakthroughs in the study of brain disorders. This review systematically summarizes the latest applications of MSI in a range of brain disorders, including Parkinson’s disease, Alzheimer’s disease, schizophrenia, and traumatic brain injury, and highlights the considerable potential of this technology for disease diagnosis and the identification of therapeutic targets. A comprehensive literature search was conducted to identify relevant studies published between 2008 and 2025. The search was performed using China National Knowledge Infrastructure (CNKI), Wanfang Data, SpringerLink, and PubMed databases. Keywords included “Alzheimer’s disease”, “Parkinson’s disease”, “traumatic brain injury”, “schizophrenia”, “DESI-MSI”, “MALDI-MSI”, “nano-MSI”, and “SIMS-MSI”, either alone or in combination. In addition to database searches, relevant articles were also identified through manual screening of reference lists from key publications to ensure comprehensive coverage of the field. Studies were included if they applied mass spectrometry imaging techniques to investigate cerebral diseases and provided spatially resolved molecular information. Articles focusing solely on mass spectrometry techniques without imaging components or without direct relevance to brain disease models were strictly excluded. The selection process followed a stepwise screening approach, in which titles and abstracts were first evaluated for relevance, followed by full-text assessment of eligible articles for final inclusion in the review.

## 2. MSI Technology and Its Characteristics

**Figure 2:**
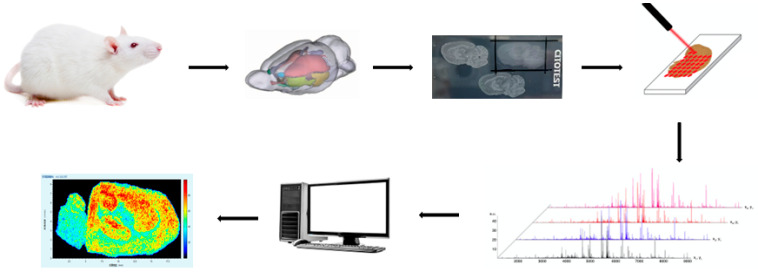
Schematic Diagram of MSI.

MSI employs various ionization methods to ionize analytes present on the surfaces of different sample types. It then conducts point-by-point scanning to acquire and record the mass spectrometric information at each spatial coordinate. Through subsequent software-based reconstruction, the signal intensities of mass spectral peaks are mapped back to their corresponding positions, generating the final molecular image (Figure 2).

### 2.1. MSI Ionization Techniques

Common ionization techniques used in MSI include matrix-assisted laser desorption/ionization (MALDI), desorption electrospray ionization (DESI), nano-desorption electrospray ionization (nano-DESI), and secondary ion mass spectrometry (SIMS). Owing to their distinct ionization principles, each technique is suited to different sample types and analytical applications, as summarized in the Table 1.

### 2.2. Characteristics of MSI

MSI offers distinct advantages in the study of brain disorders. As a label-free technique, it not only detects molecular species within tissue samples but also maps their spatial distributions. Its high spatial resolution and broad mass detection range further enable the analysis of proteins, peptides, lipids, polymers, and metabolites [28]. Compared with traditional imaging modalities, MSI avoids the need for radioactive labels or fluorescent probes and can reveal disease-related spatial heterogeneity at the molecular level, providing unique insights into the pathological mechanisms of brain disorders. For example, DESI-MSI and nano-DESI-MSI have been successfully applied to neurodegenerative disease models such as Alzheimer’s disease and Parkinson’s disease to spatially characterize lipid metabolic abnormalities and protein aggregation. Their high spatial resolution (down to the micrometer scale) allows precise discrimination of molecular differences between gray and white matter regions in brain sections [19,29]. Furthermore, when coupled with high-resolution mass analyzers (e.g., FT-ICR MS), MSI enables the identification of low-abundance metabolites in the brain, thereby enhancing the discovery of disease-related biomarkers. MSI also provides substantial advantages in the analysis of precious clinical samples, making it particularly suitable for investigating molecular dynamics in brain tumor heterogeneity, ischemia–reperfusion injury, and neuroinflammatory processes.

Despite the increasingly important role of mass spectrometry imaging (MSI) in elucidating the mechanisms of brain disorders, the technology still faces several challenges and limitations. First, substantial difficulties remain in quantitative analysis. For example, in drug distribution studies, signal intensity can vary substantially even among samples originating from the same batch due to instrumental and matrix-related factors, highlighting the necessity of robust normalization and internal standard strategies. Ref. [30], underscoring the instability of MSI in achieving accurate quantification. However, recent advances have demonstrated that the quantitative accuracy of MSI for drug distribution can be substantially improved through the use of internal standards [31] or isotopically labeled internal standards [32], tissue-matched calibration strategies [33], and pixel-wise normalization approaches [34] in both MALDI- and DESI/nano-DESI-based workflows. Second, balancing spatial resolution with detection sensitivity continues to be a major technical bottleneck. Due to sampling a smaller area or less molecules, the sensitivity is markedly decreased, resulting in the loss of detectable signals for certain low-abundance metabolites [35]. Similarly, studies using high-resolution TOF systems have shown that signal-to-noise ratios decline significantly as resolution increases [36], reflecting fundamental physical constraints at the hardware level.

Sample pretreatment and matrix effects are also major contributors to insufficient reproducibility. The crystallization uniformity of MALDI matrices has a substantial impact on signal intensity distribution, and ion suppression differences between batches can span an order of magnitude [37]. Although post-ionization strategies such as MALDI-2 improve signal intensity, they still cannot fully eliminate ionization biases arising from tissue heterogeneity [38]. In addition, when MSI is co-registered with optical or histological imaging for multimodal analysis, tissue deformation and differences in resolution can introduce positioning errors of 5–10 μm [39], thereby compromising spatial alignment accuracy. As MSI continues to extend toward single-cell and subcellular scales, challenges in sensitivity and reproducibility become even more pronounced.

Additionally, recent advances in AI-driven data interpretation have facilitated a range of computational strategies for analyzing MSI data. Promising directions include (i) automated tissue segmentation/ROI delineation from molecular profiles with multimodal validation using histology [40], (ii) supervised and deep-learning-based recognition of pathological patterns for tissue classification and grading [41,42], (iii) self-supervised representation learning to discover co-localized ion patterns without extensive manual annotations [43,44], (iv) deep-learning denoising to improve SNR and robustness of downstream analyses [45,46], and (v) multimodal integration and visualization of MSI with spatial transcriptomics/proteomics for spatial multi-omics studies [47]).

Overall, although significant progress has been achieved in mass spectrometry imaging, issues remain in quantitative capability, spatial resolution enhancement, signal standardization, and multimodal integration. These limitations continue to constrain its broader application in precision medicine and clinical practice.

## 3. Applications of MSI in Neurological Disorders

### 3.1. Parkinson’s Disease (PD)

Parkinson’s disease (PD) is a neurodegenerative disorder associated with mutations in genes such as Pink1 (PARK6), Parkin (PARK2), DJ-1 (PARK7), and α-synuclein (PARK1). Although motor impairment is the most recognizable clinical feature, patients commonly exhibit a broad spectrum of non-motor symptoms, including cognitive decline, sleep disturbances, and depression [48,49,50]. The core pathological characteristics of PD include the loss of dopaminergic neurons in the substantia nigra and the aberrant aggregation of α-synuclein, along with dysregulation of neurotransmitter signaling and calcium homeostasis [51]. The molecular pathology of PD exhibits pronounced spatial heterogeneity. Critically, the intracerebral distribution of therapeutic agents and their mode of action within vulnerable brain regions remain insufficiently understood and warrant further investigation using MSI.

In the treatment of Parkinson’s disease (PD), levodopa (L-DOPA) effectively alleviates motor symptoms; however, its long-term administration frequently leads to L-DOPA-induced dyskinesia (LID), the underlying mechanisms of which remain incompletely understood. Fridjonsdottir et al. [52] utilized MALDI-MSI to systematically characterize the spatial distribution of L-DOPA and its metabolites in the brains of non-human primate PD models during LID. Their study was the first to demonstrate brain-wide abnormal accumulation of L-DOPA and its metabolite 3-O-methyldopa (3-OMD) in LID animals, accompanied by markedly elevated dopamine (DA) levels in multiple non-striatal regions (including the hippocampus, amygdala, and bed nucleus of the stria terminalis). These DA increases exhibited a linear correlation with L-DOPA concentrations, suggesting that these regions lose effective regulatory control over DA synthesis under LID conditions. The authors further proposed that LID may arise not only from fluctuations in DA signaling but also from the central accumulation of L-DOPA itself and its direct or indirect modulation of non-striatal brain regions. This concept challenges the traditional view that LID originates solely from striatal dopaminergic dysregulation and provides a broader framework for understanding the systemic effects of L-DOPA therapy. Additionally, the concurrent elevation of L-DOPA and 3-OMD revealed by MALDI-MSI highlights potential therapeutic targets within L-DOPA metabolic pathways.

In early neurochemical studies of Parkinson’s disease (PD), the application of mass spectrometry imaging (MALDI-MSI) has progressively shifted from simple metabolite profiling to more in-depth elucidation of pathological mechanisms, revealing the spatial heterogeneity of metabolic dysregulation. In addition, the complementary integration of MALDI-MSI with in vivo ^1^H-MRS highlights the advantages of multimodal imaging approaches in enhancing both the reliability and interpretive depth of neurochemical research. Chen et al. [53] employed a combined MALDI-MSI and ^1^H-MRS strategy to characterize metabolic alterations in the medial prefrontal cortex (mPFC) of DJ-1 knockout mice. Their results showed markedly increased glutathione (GSH) levels and elevated GSH/Glu ratios in the mPFC of 9-month-old DJ-1-deficient mice, suggesting a compensatory astrocytic response to oxidative stress. This observation is consistent with elevated GSH levels reported in the prefrontal cortex of PD patients and further underscores the complexity of neurochemical regulation in transgenic models. Importantly, MALDI-MSI verified that the concentration of total creatine (tCr)—commonly used as an internal reference in ^1^H-MRS—was substantially reduced in DJ-1 knockout mice. This finding reveals potential systematic errors that may arise when relying solely on tCr or water signals for ^1^H-MRS quantification in aging or disease models. It also emphasizes the irreplaceable corrective and complementary value of MALDI-MSI in providing absolute or semi-quantitative spatial information within multi-platform validation frameworks. Despite inherent limitations related to sample preparation and technical variability, MALDI-MSI offers highly precise spatial distribution data for neurochemical species, providing essential evidence for understanding metabolic disturbances in the PD prefrontal cortex and their potential association with cognitive dysfunction.

### 3.2. Alzheimer’s Disease (AD)

Alzheimer’s disease (AD) is a neurodegenerative disorder characterized by the abnormal accumulation of Aβ plaques and tau protein. Disease progression is primarily divided into three stages: preclinical AD, mild cognitive impairment, and dementia [54]. Preclinical Alzheimer’s disease (AD), representing the earliest phase of the AD continuum, is characterized by a prolonged asymptomatic period during which individuals exhibit biological evidence of AD pathology but show no detectable cognitive or functional impairment, with everyday functioning remaining intact [55]. At present, AD drug development faces major challenges, particularly regarding drug distribution and target engagement within the brain.

**Figure 3:**
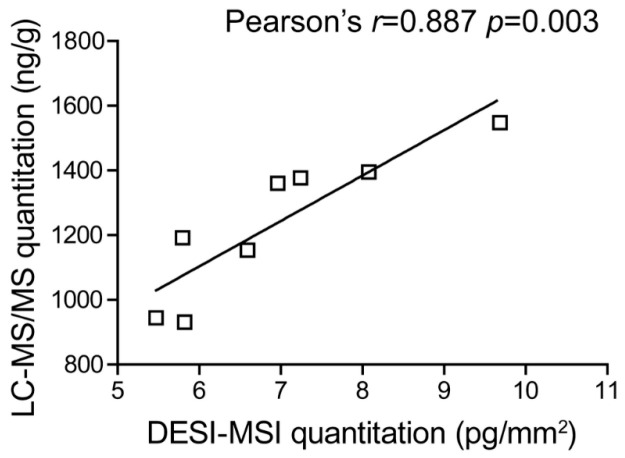
Validation of DESI-MSI versus LC-MS/MS [56]. The cubes means one-to-one corresponding data points for DC20 concentrations measured by DESI-MSI and LC-MS/MS within the same brain region.

In the context of AD drug discovery, defining the spatial distribution of candidate molecules in the brain is critical for elucidating pharmacological mechanisms and optimizing therapeutic efficacy. Mass spectrometry imaging not only visualizes regional distribution patterns but also enables highly reliable spatial pharmacokinetic analysis through multidimensional signal correction and separation strategies. Guo et al. [56] utilized desorption electrospray ionization–ion mobility spectrometry–mass spectrometry imaging (DESI-IMS-MSI) to achieve the first highly selective, semi-quantitative visualization of the novel acetylcholinesterase inhibitor Fluoropezil (DC20) in rat brain tissue. Their work demonstrated that DC20 rapidly disseminates throughout the brain within one hour post-administration and exhibits marked enrichment in the striatum. Moreover, the study established a systematic DESI-MSI optimization workflow, highlighting three major advantages for neuropharmacokinetic applications: elimination of matrix coating, in situ ion mobility-based separation, and the use of stable-isotope internal standards for region-specific signal correction. Methodologically, this study provides a broadly applicable technical framework for imaging the distribution of AD therapeutics and other central nervous system agents. The proposed three-step strategy—signal optimization, selectivity enhancement, and matrix-effect correction—offers valuable guidance not only for DESI-MSI but also for other MSI platforms. Importantly, comparative validation against conventional LC-MS/MS (Figure 3) confirms that DESI-MSI can preserve spatial information while achieving quantitative accuracy comparable to classical analytical methods. These findings firmly establish DESI-MSI as a complementary and highly informative tool for pharmacokinetic studies in neurotherapeutics.

**Figure 4:**
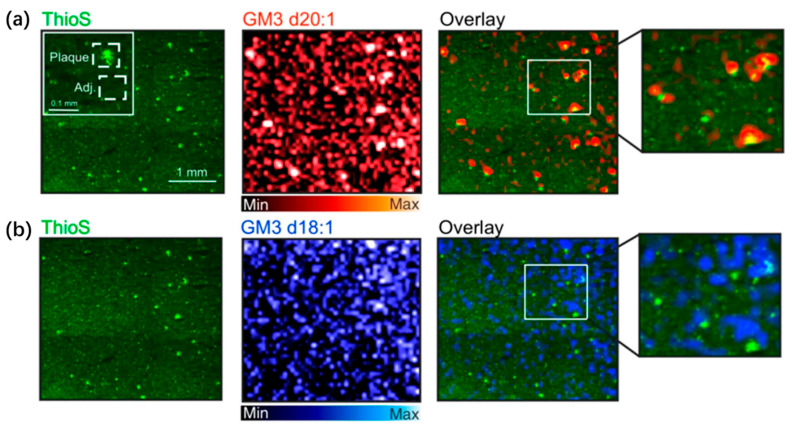
High-resolution MALDI-MSI of GM3 d20:1 (**a**) and GM3 d18:1 (**b**) in fresh-frozen AD brain tissue samples, overlaid with thioflavin S staining for amyloid β plaques [57].

In Alzheimer’s disease (AD) research, abnormalities in lipid metabolism have increasingly been recognized as key contributors to disease progression, with particular attention given to the role of gangliosides in Aβ plaque formation and tau pathology. Ollen-Bittle et al. [57] integrated MALDI-MSI with histological staining to achieve, for the first time, high-resolution visualization of the spatial distribution of gangliosides in AD patient brain tissue using the same section, thereby deepening insights into the co-localization between lipid species and pathological hallmarks. A major innovation of this study lies in its multimodal integration strategy: following MALDI-MSI acquisition, the same section was subjected to thioflavin-S staining to identify Aβ plaques, enabling precise overlay of molecular images with histopathological features. The results showed pronounced enrichment of GM3 (d18:1 and d20:1) and GM1 within plaque regions, along with significantly elevated GM3 d20:1/GM1 d20:1 ratios, suggesting a metabolic shift toward simpler gangliosides within the plaque microenvironment. In contrast, the GM1 d20:1/GM1 d18:1 ratio was markedly reduced in the entorhinal cortex and dentate gyrus molecular layer, supporting the notion of disrupted long-chain sphingoid-base ganglioside metabolism within key memory-related circuits in AD. Compared with traditional biochemical assays, MALDI-MSI offers substantial advantages, including the avoidance of tissue homogenization, preservation of spatial context, and discrimination between structurally similar lipid subtypes (e.g., d18:1 vs. d20:1). Using 40 μm resolution imaging (Figure 4), the study clearly demonstrated the strong co-localization between GM3 and Aβ plaques—an outcome that is challenging to achieve using conventional lipidomics. Moreover, the authors confirmed the feasibility of combining MALDI-MSI with histological staining in both formalin-fixed and fresh-frozen human brain tissues, providing a methodological framework for integrating digital pathology with spatial lipidomics.

Combining higher-resolution imaging with multi-omics integration offers promising opportunities to further elucidate the specific roles of lipid–protein interactions in AD pathogenesis, providing new insights for biomarker discovery and targeted therapies.

The sulfated galactose moiety of sulfatides is critical for facilitating the binding of Aβ to apoE lipoprotein particles, a process likely essential for maintaining Aβ homeostasis in the brain. Consequently, the pronounced sulfatide depletion observed in early AD may directly impair Aβ clearance, thereby promoting aggregation and deposition [58]. Zhang et al. [59] employed matrix-assisted laser desorption/ionization mass spectrometry imaging (MALDI-MSI) to systematically characterize the spatial attenuation of sulfatides and glycerophosphoinositols (GroPIns) in cognition-related brain regions, providing key spatial molecular evidence for disrupted lipid homeostasis during AD progression. By optimizing the 1,5-diaminonaphthalene (1,5-DAN) matrix spraying method, the study markedly improved lipid detection sensitivity and signal-to-noise ratio, enabling high-throughput imaging of lipids in the *m*/*z* 500–3000 range under negative ion mode. The results showed significantly reduced relative intensities of eight sulfatides (e.g., SHexCer 36:1; O_2_, SHexCer 42:2; O_2_) and two GroPIns (PI 36:4 and PI 38:4) in AD mouse brains across critical cognitive regions, including the cerebral cortex, hippocampus, and cerebellum. Notably, similar lipid depletion patterns were observed in aged wild-type mice, suggesting shared mechanisms between brain aging and AD pathology. This work highlights sulfatides and GroPIns as potential biomarkers for AD and emphasizes the unique capabilities of MALDI-MSI for label-free, in situ, multi-target lipid analysis, offering a robust technical approach for investigating lipid metabolic dysregulation in brain aging and neurodegenerative diseases.

### 3.3. Schizophrenia

The etiology and pathogenesis of schizophrenia are complex, involving multiple pathways, including genetic, neurodevelopmental, neurotransmitter, and immune–inflammatory mechanisms. Recent studies increasingly indicate that the disorder is not merely a disruption of neurochemical signaling but is also accompanied by widespread molecular metabolic and post-translational modification abnormalities, particularly in glycosylation, lipid metabolism, and oxidative stress pathways [60]. By enabling label-free, spatially resolved detection, MSI allows in situ imaging of neurotransmitters, lipids, and glycans while preserving tissue morphology, providing a novel approach for investigating metabolic dysregulation and brain region-specific molecular distributions in schizophrenia.

In neurobiological studies of schizophrenia, abnormal glycosylation has emerged as a potential molecular mechanism contributing to disease pathophysiology [61]. Analyses of postmortem brain tissues have reported aberrant glycosylation patterns in schizophrenia, including altered glycosylation of proteins involved in inhibitory and excitatory neurotransmission, such as glutamate transporters and γ-aminobutyric acid (GABA) receptors, as well as dysregulated expression of glycosyltransferases [61,62]. These observations were primarily derived from biochemical, proteomic, and molecular studies rather than imaging-based approaches. Mass spectrometry imaging (MSI) has been developed as a complementary, label-free technique for spatially characterizing the brain glycome. As reviewed by Hasan et al., MSI enables the simultaneous visualization of multiple glycans directly within tissue sections, overcoming limitations of conventional lectin- or antibody-based methods related to specificity, tissue penetration, and multiplexing capacity. When combined with on-tissue enzymatic digestion strategies, such as PNGase F treatment, MSI allows high-throughput, in situ release and imaging of N-glycans while preserving tissue architecture. This approach reveals pronounced regional heterogeneity in glycan distributions across brain regions, enabling the detection of dozens of N-glycans within a single brain section—an analysis that remains challenging using traditional immunohistochemical techniques. However, because glycans are enzymatically cleaved from their parent glycoproteins prior to MSI analysis, this strategy does not retain direct information regarding the specific proteins to which the detected glycans were originally attached [61,63].

In the future, with advancements in matrix-assisted laser desorption/ionization secondary ion mass spectrometry (MALDI-2 [64]) and ultra-high-resolution mass spectrometers (e.g., 21 T FT-ICR), MSI is expected to further improve sensitivity, resolution, and coverage for glycan detection. These enhancements are anticipated to play a pivotal role in elucidating glycosylation regulatory mechanisms in neuropsychiatric disorders such as schizophrenia.

**Figure 5:**
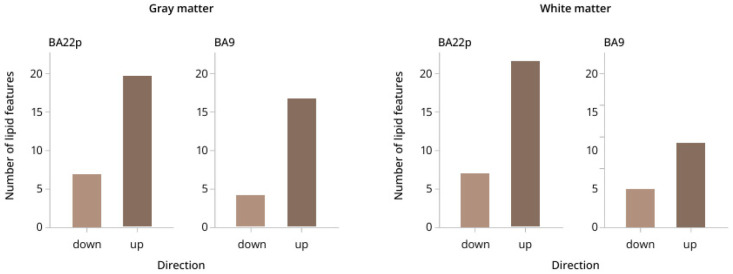
Lipid distribution differences in distinct brain regions of schizophrenia patients [65].

Moreover, MSI’s integrated capabilities for multi-brain-region comparison, tissue-sectional analysis, and lipid spatial visualization provide a novel dimension for investigating brain region-specific molecular pathology in schizophrenia. Osetrova et al. [65] employed MALDI-MSI to perform high-spatial-resolution comparative lipid distribution analyses across two functionally distinct brain regions: the dorsolateral prefrontal cortex (Brodmann area 9, BA9) and the posterior superior temporal lobe (Brodmann area 22 posterior, BA22p). This study conducted region-specific lipidomics profiling of both gray and white matter without disrupting tissue integrity, revealing that schizophrenia-associated lipid alterations exhibit pronounced brain region selectivity and tissue-type specificity. The results demonstrated that lipid expression changes were especially prominent in the white matter of BA22p, with approximately twice as many differentially altered lipids compared to BA9 (31 vs. 17), whereas gray matter alterations were largely consistent between the two regions (Figure 5). These findings underscore spatial heterogeneity that conventional homogenized lipidomics cannot resolve. Importantly, despite differences in the extent of lipid alterations across regions, trends at the lipid class level (e.g., phosphatidylcholines, sphingomyelins) were highly correlated in gray matter, indicating that major pathological changes retain a degree of conservation. Notably, acylcarnitines were consistently downregulated in both gray and white matter across the two brain regions, highlighting their potential as lipid biomarkers associated with schizophrenia.

### 3.4. Traumatic Brain Injury (TBI)

Traumatic brain injury (TBI) is a complex pathological process characterized by structural and functional damage to brain tissue induced by external forces, commonly arising from traffic accidents, falls, violence, or blast exposures [66]. Clinically, TBI exhibits marked heterogeneity, ranging from mild concussion to severe contusion and laceration, each resulting in varying degrees of neurological dysfunction. Epidemiological data indicate that TBI is a leading cause of death and long-term disability worldwide, accounting for more than 30% of all trauma-related fatalities [67]. The pathological progression of TBI generally comprises two stages: primary mechanical injury and secondary molecular-level damage. The former involves cellular disruption and blood–brain barrier compromise due to direct physical impact, while the latter encompasses a cascade of biochemical responses, including oxidative stress, inflammatory reactions, mitochondrial dysfunction, and lipid metabolism disturbances [68,69,70]. These secondary processes manifest as significant alterations in lipid and metabolite profiles, which in turn affect neuronal membrane stability, energy metabolism, and neurotransmitter cycling [71]. Therefore, precisely analysing the molecular reprogramming dynamics following TBI is crucial for elucidating secondary neuropathology mechanisms and identifying potential therapeutic targets.

In TBI research, lipid metabolic dysregulation is considered a critical mediator of secondary neuroinflammation and neurodegeneration. Mallah et al. [72] employed MALDI-MSI to systematically investigate the spatiotemporal dynamics of lipid distribution across multiple brain regions at post-injury time points of 1, 3, 7, and 10 days in a controlled cortical impact rat model. A key strength of this study is its ability to simultaneously acquire high-spatial-resolution lipid images across the whole brain, encompassing lipid species from the low-mass range (<600 Da) to the high-mass range (600–900 Da), thereby overcoming the limitations of prior studies that were often restricted to the injury core.

Unsupervised spatial segmentation analysis revealed unique lipid expression clusters within the injury region, and subsequent receiver operating characteristic analysis identified 82 injury-specific lipid signals. Notably, within the low-mass range, acylcarnitines—particularly palmitoylcarnitine (*m*/*z* 400.35)—peaked at day 3 post-injury and were predominantly localized to the injury periphery rather than the core.

More critically, immunofluorescence staining of serial sections from the same brain slice revealed colocalization of palmitoylcarnitine with CX3CR1-high resident microglia, whereas no significant colocalization was observed in regions infiltrated by Mac-2-positive macrophages. This finding suggests that palmitoylcarnitine may participate in microglia-mediated formation and containment of the injury boundary. Additionally, significant palmitoylcarnitine expression was detected in the ipsilateral substantia nigra at day 3 post-injury, providing early molecular-level evidence linking TBI to an increased risk of Parkinson’s disease and highlighting MALDI-MSI’s unique capability for revealing trans-synaptic propagation of pathology in distant brain regions.

MALDI-MSI offers comprehensive advantages for identifying elusive lipid biomarkers, elucidating their cellular origins, and characterizing their spatiotemporal pathological dynamics, thus providing new insights and potential targets for therapeutic intervention in secondary injury following TBI.

Moreover, metabolic reprogramming is increasingly recognized as a key driver of disease progression in TBI. Siciliano et al. [73] employed a targeted approach using atmospheric pressure matrix-assisted laser desorption/ionization mass spectrometry imaging (AP-MALDI-MSI) to systematically map the spatial distribution of small-molecule metabolites in mouse brain tissue post-TBI. Analysis of brain sections at day 21 post-injury, using imaging with a spatial resolution of 100 μm, identified four metabolites—alanine, lysine, histidine, and inosine—with overall elevated expression in the TBI model, particularly in the ipsilateral (injured) hemisphere.

Region-of-interest analysis focused on the thalamus revealed significant increases in arginine, lysine, histidine, and inosine within the ipsilateral thalamus, while glutamate and N-acetylaspartate exhibited pronounced decreases. Adjacent tissue sections were validated by LC-MS/MS, confirming the reliability of metabolite identification and quantification. Interestingly, the observed glutamate reduction contrasts with reports of elevated glutamate in acute-phase studies, indicating that metabolic changes are both time- and model-dependent. These findings underscore the importance of MSI in capturing dynamic metabolic adaptations following TBI, demonstrate its sensitivity in detecting subacute-phase metabolic abnormalities, and provide a novel spatial perspective for understanding long-term neurometabolic dysregulation associated with TBI.

### 3.5. Other Applications

Besides the four conditions discussed above, the application of MSI is rapidly expanding across a broad spectrum of brain pathologies. These examples are briefly introduced to illustrate the broader applicability of MSI, rather than to provide an exhaustive discussion. In neuroinflammation, MSI enables the delineation of spatial trajectories associated with microglial activation-driven lipid remodeling and metabolic reprogramming, thereby uncovering dynamic relationships between inflammatory phenotype transitions (e.g., shifts toward glycolysis) and membrane lipid signaling [74,75,76]. Pagel et al. [74] revealed LPS-induced disruptions in lipid pathways using MALDI-MSI at the cellular level, while Di Carli et al. [75] further demonstrated the early-stage co-localization of sphingolipids and oxidized lipids through multimodal MSI. Together with emerging insights into the “metabolism–inflammation coupling” between microglial metabolic states and neurodegenerative progression [76], these results highlight the unique strengths of MSI in characterizing molecular regulation within the inflammatory microenvironment.

In epilepsy research, MALDI-MSI has recently been explored for pathological interrogation of surgically resected tissue from patients with refractory epilepsy. Applying MALDI-MSI to classical focal cortical dysplasia (FCD IIb) specimens, researchers distinguished lesion cores from surrounding tissue through label-free in situ molecular profiling, identifying peptide fingerprint signatures with diagnostic potential as well as molecular maps of lesion boundaries [77]. Although the application of MSI to epilepsy pathology remains at an early stage, its unique capability for direct molecular measurement while retaining spatial architecture offers substantial potential for both pre- and post-surgical tissue evaluation, including boundary delineation and residual lesion detection.

In Huntington’s Disease (HD) Research, a study employed MALDI-MSI to conduct spatial proteomic imaging on brain tissue from YAC128 transgenic mice, identifying 22 differentially expressed proteins. Immunohistochemistry further validated the regional distribution changes of GFAP, a marker for neuronal loss [78]. This research underscores the ability of MSI to provide dual-dimensional information, encompassing both molecular perturbations and spatial localization, which is crucial for studying neurodegenerative diseases. Such applications offer unique insights into the progression of HD and the development of biomarkers.

In Addiction Research, one investigation used MALDI-MSI to map regional variations in peptide and protein expression in the reward circuitry of rat brains, including the hippocampus, prefrontal cortex, amygdala, and nucleus accumbens, following combined cocaine and ethanol administration. This analysis revealed the remodeling of molecular networks in the brain due to poly-drug use [79]. DESI-MSI demonstrates that morphine, cocaine, and amphetamine exposure induces region-specific remodeling of brain lipids [80]. Infrared matrix-assisted laser desorption electrospray ionization (IR-MALDESI)-MSI enabled spatial distributions imaging of morphine in mouse brains, facilitating in vivo assessment of opioid exposure in real tissue [81]. Meanwhile, TOF-SIMS imaging linked phospholipid abnormalities induced by cocaine exposure and the reversal effects of methylphenidate to behavioral phenotypes, providing spatial molecular evidence to elucidate mechanisms and evaluate potential interventions [82].

## 4. Conclusions

In conclusion, mass spectrometry imaging (MSI) offers substantial advantages in the study of brain disorders. It enables the simultaneous detection of multiple molecules in a label-free manner, providing a direct visualization of their spatial distribution within brain regions, thus presenting new tools for unraveling complex pathological processes. In research on various diseases, including Parkinson’s disease (PD), Alzheimer’s disease (AD), schizophrenia, and traumatic brain injury (TBI), MSI has uncovered critical alterations in neurotransmitters, lipids, and metabolites, providing novel insights into disease mechanisms, as summarized across representative studies in Table 2.

However, the technology still faces several challenges, such as limited quantitative capabilities, constraints in spatial resolution, low sensitivity for detecting low-abundance molecules, and a limited supply of clinical samples. These limitations hinder the broader applicability of research findings and their clinical translation. Future studies should address key issues such as the establishment of quantitative standards, the balance between spatial resolution and sensitivity, the standardization of sample preparation, and the development of multimodal fusion analysis. With the concurrent advancement of high-resolution mass spectrometry, single-cell imaging, and AI-driven data interpretation, MSI holds great promise to play an increasingly pivotal role in elucidating the molecular networks of brain disorders, discovering novel biomarkers, and supporting precision diagnosis and personalized therapies in clinical practice.

## Figures and Tables

**Table 1:** Principles, Applications, and Advantages/Disadvantages of Four Common Ion Sources.

Ion Source	Ionization Principle	Suitable Molecules
Matrix-Assisted Laser Desorption/Ionization (MALDI)	A matrix, such as α-cyano-4-hydroxycinnamic acid (CHCA) or 2,5-dihydroxybenzoic acid (DHB), is uniformly applied to the sample surface and co-crystallized with the analyte. Subsequently, a high-spatial-resolution ultraviolet laser is focused onto predetermined pixel locations, enabling the matrix to absorb laser energy and facilitate desorption and ionization of the analyte [11].	Proteins [12], peptides [12], phospholipids [13], carbohydrates [14], metabolites [15]
Desorption Electrospray Ionization (DESI)	An atomized gas is combined with a charged solvent containing a high proportion of organic components (e.g., acetonitrile/water/formic acid) via a capillary to generate an electrospray, which is directed onto the sample surface. Upon impact, these high-velocity charged droplets desorb and extract surface analyte molecules through a mechanism known as “droplet pick-up.” In this process, droplets form a thin liquid film on the surface, dissolve the analytes, and are subsequently disrupted by incoming droplets, generating secondary charged droplets that contain the analytes. During their migration toward the mass spectrometer inlet, these droplets undergo desolvation and Coulombic fission, ultimately producing gas-phase ions [16].	Phospholipids [17], metabolites [18]
Nano-DESI (nano-DESI)	The key feature of this technique is a probe composed of two micrometer-scale capillaries. During operation, the primary capillary delivers solvent to the probe tip, forming a microliter-scale dynamic liquid bridge at the interface with the sample surface. This liquid bridge selectively desorbs and extracts soluble molecules from the surface. A secondary nano-spray capillary then rapidly aspirates the analyte-containing solution and directs it toward the mass spectrometer inlet. Applying a high voltage to the tip of the nano-spray capillary generates charged droplets, thereby enabling soft ionization of the sample [19].	Phospholipids [20], metabolites [21], peptides [22,23]
Secondary Ion Mass Spectrometry (SIMS)	A high-energy primary ion beam generated by a liquid metal ion source (e.g., Bi^+^ or Bi^3+^) is focused onto the sample surface. Through collision-induced energy transfer during the sputtering process, surface atoms or molecules are emitted as secondary ions. These positively or negatively charged secondary ions are subsequently extracted and accelerated into a field-free flight tube. The ions travel toward the detector at velocities determined by their mass-to-charge ratio (*m*/*z*), with flight times proportional to the square root of the ion mass, thereby enabling precise mass analysis [24].	Phospholipids [25], metabolites [26], peptides [27]

**Table 2:** Overview of MSI-based discoveries highlighted in this review, their spatial molecular features, and clinical translation challenges.

MSI-Based Discovery (from This Review)	Spatial Molecular Feature	Potential Clinical Relevance	Major Non-Technical Barriers to Translation
TBI	Time-dependent and spatially restricted accumulation of acylcarnitines (e.g., palmitoylcarnitine) at the injury periphery, with microglia-associated localization	Potential prognostic relevance for secondary injury progression and neuroinflammatory dynamics	Lack of prospective human validation; limited correlation with functional or long-term clinical outcomes; need for conversion into standardized, targeted analytical assays
Schizophrenia	Downregulation of acylcarnitines and class-level lipid alterations across gray and white matter in distinct cortical regions	Disease-associated molecular signatures with potential relevance for mechanistic stratification	Predominant reliance on postmortem tissue; confounding effects of medication and disease heterogeneity; absence of longitudinal and treatment-response studies
AD	Plaque-associated ganglioside enrichment and region-specific sulfatide depletion	Pathophysiological indicators with potential relevance for disease stratification	Lack of validated quantitative thresholds; limited linkage to clinical staging and outcomes; restricted accessibility of comparable tissue from living patients

## Data Availability

No new data were created or analyzed in this study.

## Abbreviations

The following abbreviations are used in this manuscript:

MSI	Mass Spectrometry Imaging
MALDI	Matrix-Assisted Laser Desorption/Ionization
DESI	Desorption/Ionization Electrospray
DALYs	Disability-Adjusted Life Years
SIMS	Secondary Ion Mass Spectrometry
CHCA	α-cyano-4-hydroxycinnamic acid
DHB	2,5-dihydroxybenzoic acid
FT-ICR MS	Fourier Transform Ion Cyclotron Resonance Mass Spectrometry
PD	Parkinson’s Disease
L-DOPA	Levodopa
LID	L-DOPA-induced dyskinesia
3-OMD	3-O-Methyldopa
DA	Dopamine
1H-MRS	Hydrogen proton Magnetic Resonance Spectroscopy
AD	Alzheimer’s Disease
IMS	Ion Mobility Spectrometry
GABA	Gamma-Aminobutyric Acid
TBI	Traumatic Brain Injury
FCD	Focal Cortical Dysplasia
HD	Huntington’s Disease
IR-MALDESI	Infrared-Matrix-Assisted Laser Desorption Electrospray Ionization
TOF-SIMS	Time of Flight Secondary Ion Mass Spectrometry
